# Psychiatric adverse events associated with GLP-1 receptor agonists: a real-world pharmacovigilance study based on the FDA Adverse Event Reporting System database

**DOI:** 10.3389/fendo.2024.1330936

**Published:** 2024-02-06

**Authors:** Wei Chen, Peishan Cai, Wenbin Zou, Zhiwen Fu

**Affiliations:** ^1^ Department of Pharmacy, Union Hospital, Tongji Medical College, Huazhong University of Science and Technology, Wuhan, Hubei, China; ^2^ Department of Thoracic Surgery, Tongji Hospital, Tongji Medical College, Huazhong University of Science and Technology, Wuhan, Hubei, China

**Keywords:** glucagon-like peptide-1 receptor agonists, psychiatric adverse events, disproportionality analyses, FAERS database, pharmacovigilance study

## Abstract

**Background:**

Glucagon-like peptide-1 receptor agonists (GLP-1 RAs) are widely used due to their profound efficacy in glycemic control and weight management. Within real-world contexts, the manifestation of certain psychiatric adverse events (AEs) has been observed, which is potentially linked to the administration of GLP-1 RAs. The objective of this study was to undertake a comprehensive investigation and characterization of the psychiatric AEs associated with GLP-1 RAs.

**Methods:**

We retrieved reports of AEs associated with treatment with GLP-1 RAs during the period from the first quarter (Q1) of 2004 to Q1 2023 from the FDA Adverse Event Reporting System (FAERS) database. Descriptive analysis was performed to examine the clinical characteristics and time to onset of the psychiatric AEs caused by GLP-1 RAs. Moreover, disproportionality analyses were performed using the reporting odds ratio (ROR) to identify GLP-1 RA-related psychiatric AEs.

**Results:**

A total of 8,240 reports of psychiatric AEs were analyzed out of 181,238 AE reports with treatment with GLP-1 RAs. Among these cases, a higher percentage was represented by women compared to men (65.89% *vs*. 30.96%). The median age of these patients was 56 years, with an interquartile range (IQR) of 48–67 years, based on data available in 286 case reports. This study showed that the median time to onset of the overall GLP-1 RA-related AEs was 31 days (IQR = 7–145.4 days), which varied among GLP-1 RA regimens. Specifically, exenatide had a significantly longer onset time at 45 days (IQR = 11–213 days), with statistically significant differences from the onset times of the other five GLP-1 RAs (*p*< 0.0001). Moreover, eight categories of psychiatric AEs, namely, nervousness (ROR = 1.97, 95% CI = 1.85–2.11), stress (ROR = 1.28, 95% CI = 1.19–1.38), eating disorder (ROR = 1.57, 95% CI = 1.40–1.77), fear of injection (ROR = 1.96, 95% CI = 1.60–2.40), sleep disorder due to general medical condition—insomnia type (ROR = 2.01, 95% CI = 1.60–2.52), binge eating (ROR = 2.70, 95% CI = 1.75–4.16), fear of eating (ROR 3.35, 95% CI = 1.65–6.78), and self-induced vomiting (ROR = 3.77, 95% CI = 1.77–8.03), were defined as GLP-1 RA-related psychiatric AEs through disproportionality analysis.

**Conclusion:**

Our findings demonstrate a significant association between GLP-1 RAs and the development of specific psychiatric AEs. Despite the observational nature of this pharmacovigilance study and the inherent limitations of the FAERS database, our preliminary findings in this work could provide a better basis for understanding the potential psychiatric AEs that may occur with GLP-1 RA treatment, assisting clinicians to focus on these AEs and provide early intervention for optimal risk management.

## Introduction

Diabetes has been one of the fastest growing public health issues worldwide in recent decades. The year 2021 witnessed a staggering 529 million diagnosed cases of diabetes, with projections indicating a substantial increase to over 1.31 billion cases by the year 2050 (1). Glucagon-like peptide-1 receptor agonists (GLP-1 RAs) comprise a therapeutic class influencing glycemic control through multiple mechanisms, including increasing glucose-dependent insulin secretion, delayed gastric emptying, reduced postprandial glucagon, and decreased food intake (2, 3). Numerous studies have highlighted the distinctive benefits of GLP-1 RAs in diabetes management, showcasing a reduced risk of hypoglycemia, the capacity to restore β-cell function, and the potential to enhance cardiovascular outcomes, particularly in high-risk populations (4, 5). Recent studies have also shown their potential to penetrate the blood–brain barrier and access multiple central nervous system nuclei in the brain to drive neuroprotective responses (6). In addition, GLP-1 RAs have demonstrated noteworthy impacts on weight management among individuals with obesity with and without diabetes (7).

GLP-1 RAs are experiencing expanded global utilization and favorable market potential. According to the financial reports by Novo Nordisk, the business revenue of GLP-1 RAs has reached 34,653 million Danish krone (DKK) in the first quarter of 2023 (8). With the widespread use of GLP-1 RAs, reports of adverse events (AEs) have been increasing. Following a potential thyroid cancer risk alert from the European Medicines Agency (EMA), GLP-1 RAs have recently been investigated for a possible suicide risk (9). The administration of semaglutide in rodents at clinically relevant exposures has been observed to induce thyroid cell tumors in a dose-dependent and treatment time-dependent manner. However, extensive clinical trial data have not demonstrated a causal association between GLP-1 RAs and suicidal or self-harming intent (10). Among the numerous published randomized controlled trials, only a limited number of studies have reported on psychiatric AEs associated with GLP-1 RAs (11). Currently, real-world research attention on the adverse effects of GLP-1 has focused on gastrointestinal adverse effects, metabolic ketoacidosis, and tumor-associated AEs (12–14). Moreover, real-world data analyzing psychiatric AEs with GLP-1 RA treatment and comparisons of the psychiatric disorder differences among specific GLP-1 RA agents remain scarce.

The FDA Adverse Event Reporting System (FAERS) is a robust pharmacovigilance database that contains real-world data on AE reporting (15). The extensive size of FAERS enhances its capability to detect potential AE signals, serving as a valuable tool for identifying even rare potential AEs. In addition, FAERS is equipped to capture various clinical characteristics associated with specific AEs, including details such as the history of AEs and the prognosis of patients. In this context, we intended to conduct a disproportionality analysis utilizing FAERS data. The objectives were to comprehensively evaluate the psychiatric AEs associated with GLP-1 RAs and to characterize the clinical features observed in the reported cases. Moreover, the differences among different GLP-1 RAs were examined.

## Methods

### Data sources

FAERS is an openly accessible pharmacovigilance database documenting AEs, medication errors, and product quality complaints reported spontaneously by people of diverse occupations worldwide (14). Utilizing the extensive real-world data within FAERS, we performed a pharmacovigilance study to examine the association between GLP-1 RAs and psychiatric AEs. The FAERS database comprised seven files, namely, demographic and administrative details (coded as DEMO), medication information (DRUG), reports of adverse events (REAC), patient outcomes (OUTC), reporting sources such as healthcare professionals or consumers (RPSR), treatment initiation and discontinuation dates (THER), and indications for medication use (INDI), as described previously (16). Our study extracted all reports from the first quarter (Q1) of 2004 to Q1 2023, with a total of 19,494,698 reports retrieved from the FAERS database during the study period. To remove duplicate records, a systematic de-duplication process was implemented based on two criteria (17): i) When the report identification code (CASEID) was identical across entries, the case with the most recent FDA receipt date (FDA_DT) was retained. ii) For reports with identical CASEID and FDA_DT, the entry with the higher primary identifier code (PRIMARYID) was selected. As a result, 16,529,887 reports were retained for final analysis in the present study.

### Data extraction

In order to acquire AE reports data treated with GLP-1 RAs, we identified the AEs of GLP-1 RAs in the DRUG files using the generic and brand names of the FDA-approved GLP-1 RAs, specifically exenatide (BYETTA, BYDUREON), liraglutide (VICTOZA, SAXENDA), lixisenatide (ADLYXIN, SOLIQUA), dulaglutide (TRULICITY), semaglutide (OZEMPIC, RYBELSUS, WEGOVY), and tirzepatide (MOUNJARO). The specific marketing approval dates for these GLP-1 RAs are shown in **Supplementary Table S1**. The target drugs for our study were identified in the DRUG file, encompassing both generic and trade names as previously mentioned, and the role_cod was chosen as primary suspect (PS) drugs.

Since all of the AEs reported in the FAERS database are categorized using preferred term (PT) codes based on the Medical Dictionary for Regulatory Activities (MedDRA), we extracted the PTs related to mental disorders in MedDRA (version 24.0) according to the system organ classification (SOC) of “psychiatric disorders.” Accordingly, we analyzed these PTs with the GLP-1 RAs classified in the psychiatric disorders SOC (SOC: 10037175). We conducted calculations and analysis on the demographic factors (gender and age) and the reporting characteristics (reporting country, reporting year, occupation of reporters, and drug indications) associated with the psychiatric AEs occurring during treatment with GLP-1 RAs. Furthermore, we statistically analyzed the outcomes (death, life-threatening, hospitalization, disability, congenital anomaly, required intervention to prevent, and other medical events) with the different GLP-1 RA treatments and determined the time to onset of the psychiatric AEs induced by each GLP-1 RA. Time to onset was defined as the time interval between the initiation of GLP-1 RAs and the occurrence of an AE. When calculating the onset time, we only selected data with an onset time greater than 0 days. Reports with incorrect dates (i.e., time of dosing later than the time of event) and missing dates were not included.

### Signal mining

Based on the FAERS database, we employed a disproportionality analysis model to identify potential signals of psychiatric AEs caused by GLP-1 RAs. When a target drug is more likely to induce a target AE than all other drugs, it usually receives a higher score due to disproportionality and a higher correlation signal (18).

With data from the entire FAERS database as a background, the reporting odds ratio (ROR) was used to compare the odds of reporting drug-specific related AEs with all other AEs. In examining the association between treatment with GLP-1 RAs and the occurrence of psychiatric AEs, we used the ROR to detect psychiatric AE signals in the GLP-1 RA reports. The potential association between psychiatric AEs and GLP-1 RAs was assessed by comparing the psychiatric AE reports for GLP-1 RAs with the complete FAERS database, performing a disproportionality analysis, and calculating the ROR. Specific formulas for calculating the ROR and the 95% confidence intervals (CIs) are shown below.


$$\text{ROR}=\;\frac{a/c}{b/d}$$

$$95\%\;\text{CI}=\;e^{\text{ln}\left(\text{ROR}\right)\pm 1.96\sqrt{\frac{1}{a}+\frac{1}{b}+\frac{1}{c}+\frac{1}{d}}}$$

where *a* is the number of targeted case reports involving GLP-1 RAs; *b* is the number of other case reports involving GLP-1 RAs; *c* is the number of targeted case reports involving the other drugs in the full database; and *d* is the number of other case reports involving the other drugs in the full database.

Among the obtained psychiatric AE reports, GLP-1 RA-related psychiatric AEs were defined as the AEs whose total report number was no less than 5 and whose lower limit of the 95% CI of the ROR (ROR05) was greater than 1. It is noteworthy that, in the statistical analysis, the total number of reports was typically defined as a minimum of 3. However, given our comprehensive examination of the class of six GLP-1 RAs, we have established a minimum threshold of 5 reports to mitigate the potential false-positive signals. Based on the above rules, we screened the reports of the GLP-1 RA-related psychiatric AEs and further analyzed the results. SAS (version 9.4) was used to analyze the data.

## Results

### Psychiatric AEs among patients who received GLP-1 RAs in the FAERS database: Q1 2004–Q1 2023

**Figure 1:**
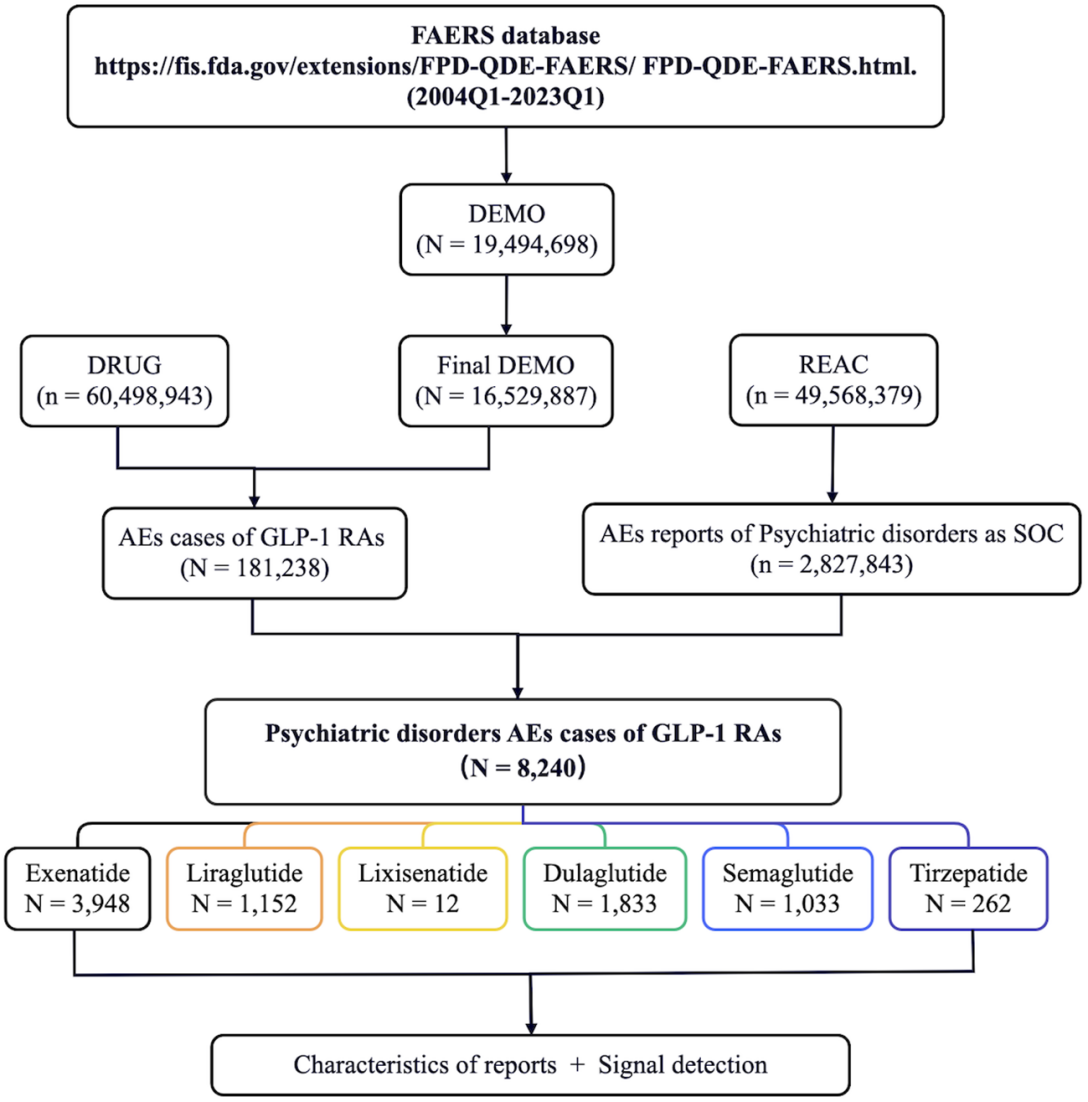
Flow diagram of the screening of psychiatric adverse events (AEs) of glucagon-like peptide-1 receptor agonists (GLP-1 RAs) from the FDA Adverse Event Reporting System (FAERS) database.

Firstly, we extracted the reports regarding psychiatric AEs among patients who received GLP-1 RAs from the FAERS database between Q1 2004 and Q1 2023. A total of 181,238 AE reports with treatment with GLP-1 RAs were documented in the FAERS database, of which 8,240 were psychiatric AEs. Psychiatric AEs accounted for 4.55% (8,240/181,238) of the total number of AEs associated with GLP-1 RAs. Of these, 3,948 (47.91%) psychiatric AE cases were for exenatide, 1,152 (13.98%) for liraglutide, 12 (0.15%) for lixisenatide, 1,833 (22.25%) for dulaglutide, 1,033 (12.54%) for semaglutide, and 262 (3.18%) were for tirzepatide (**Figure 1**). **Supplementary Table S1** provides information on the time to market of the various GLP-1 RAs. Disparities in the quantity and percentage of the documented psychiatric AEs among different GLP-1 RAs could potentially be associated with factors such as their duration in the market (e.g., more time for AEs to be reported into FAERS for medications that have been available for longer), clinical utilization, and specific drug characteristics.

The occurrence of psychiatric AEs on the SOC level also varied among treatments with different GLP-1 RAs. With tirzepatide treatment, the case number of psychiatric AEs as a percentage of all the AEs reported was 2.71%, while psychiatric AEs were recorded in 5.82% of all AEs in cases treated with semaglutide. The contribution of psychiatric AEs in GLP-1 RAs is not prominent compared with the background frequency of the SOC level, but with the dramatic increase in the number of GLP-1 RAs used, psychiatric AEs remain a notable proportion of the overall AEs associated with GLP-1 RAs (**Table 1**).

**Table 1:** Signal detection for adverse events (AEs) of glucagon-like peptide-1 receptor agonists (GLP-1 RAs) with psychiatric AEs (pAEs) in the FDA Adverse Event Reporting System (FAERS) database from 2004 to 2023.

	With psychiatric AE cases	Without psychiatric AE cases	All AE cases	pAEs/all AEs (%)
Overall	8,240	172,998	181,238	4.55
Exenatide	3,948	68,429	72,377	5.45
Liraglutide	1,152	27,828	28,980	3.98
Lixisenatide	12	299	311	3.86
Dulaglutide	1,833	50,325	52,158	3.51
Semaglutide	1,033	16,708	17,741	5.82
Tirzepatide	262	9,409	9,671	2.71

### Descriptive analysis

The statistical results of the clinical characteristics of the psychiatric AE reports for GLP-1 RAs (*n* = 8,240) are displayed in **Table 2**. Among these cases, women (*n* = 5,429, 65.89%) accounted for a larger proportion than men (*n* = 2,551, 30.96%). Of the 286 AE reports with available information on age, the median age of patients was 56 years [interquartile range (IQR) = 48–67]. North America exhibited the highest incidence of reported psychiatric AEs (*n* = 7,673, 93.12%), which could potentially be attributed to its leading number of prescriptions for GLP-1 RAs, followed by Europe (*n* = 299, 3.63%) and Asia (*n* = 137, 1.66%). Compared to the number of psychiatric AEs reported in 2021 (*n* = 935) and 2022 (*n* = 893), the psychiatric AEs of GLP-1 RAs reported in Q1 alone in 2023 reached a whopping 411 cases, demonstrating the growing clinical use of GLP-1 RAs. The majority of reports were submitted by consumers (*n* = 6,577, 79.82%) and physicians (*n* = 694, 8.42%). The primary indications for GLP-1 RAs, in theory, encompassed diabetes and weight control, accounting for nearly 100% of cases. Our analysis of the collected data revealed that psychiatric AEs were most frequently reported in relation to diabetes (*n* = 5,286, 64.15%) and weight control (*n* = 332, 4.03%). However, it is important to note that, due to potential underreporting by the informants, there were also instances of unknown indications (*n* = 2,281, 27.68%). The most frequent AE outcomes were those that resulted in hospitalization (*n* = 883, 10.72%) or other medical events (*n* = 1,064, 12.91%).

**Table 2:** Characteristics of glucagon-like peptide-1 receptor agonist (GLP-1 RA)-associated psychiatric disorders: adverse event (AE) reports sourced from the FDA Adverse Event Reporting System (FAERS) database (Q1 2004–Q1 2023).

Characteristics	All GLP-1 RAs (*N* = 8,240)		Exenatide (*n* = 3,948		Liraglutide (*n* = 1,152)		Lixisenatide (*n* = 12)		Dulaglutide (*n* = 1,833)		Semaglutide (*n* = 1,033)		Tirzepatide (*n* = 262)	
Demographics														
Gender	*n*	%	*n*	%	*n*	%	*n*	%	*n*	%	*n*	%	*n*	%
Women	5,429	65.89	2,709	68.62	775	67.27	4	33.33	1,098	59.90	655	63.41	188	71.76
Men	2,551	30.96	1,200	30.40	350	30.38	6	50.00	617	33.66	330	31.95	48	18.32
Unknown	260	3.16	39	0.99	27	2.34	2	16.67	118	6.44	48	4.65	26	9.92
Age	*n*	%	*n*	%	*n*	%	*n*	%	*n*	%	*n*	%	*n*	%
Known	286	3.47%	21	0.53%	25	2.17%	1	8.33%	24	1.31%	114	11.04%	101	38.55%
Unknown	7,954	96.53%	3,927	99.47%	1,127	97.83%	11	91.67%	1,809	98.69%	919	88.96%	161	61.45%
Median (IQR)	56 (48, 67)		63 (57, 69)		53 (44, 65)				66 (60, 71)		59 (45, 72)		53 (43, 60)	
Reporting characteristics														
Reporting region	*n*	%	*n*	%	*n*	%	*n*	%	*n*	%	*n*	%	*n*	%
North America	7,673	93.12	3,766	95.39	1,004	87.15	6	50.00	1,692	92.31	945	91.48	260	99.24
Europe	299	3.63	94	2.38	74	6.42	2	16.67	74	4.04	55	5.32	0	0.00
Asia	137	1.66	47	1.19	30	2.60	2	16.67	47	2.56	11	1.06	0	0.00
South America	68	0.83	18	0.46	29	2.52	2	16.67	9	0.49	9	0.87	1	0.38
Oceania	47	0.57	16	0.41	8	0.69	0	0.00	10	0.55	13	1.26	0	0.00
Africa	7	0.08	3	0.08	3	0.26	0	0.00	0	0.00	0	0.00	1	0.38
Unknown	9	0.11	4	0.10	4	0.35	0	0.00	1	0.05	0	0.00	0	0.00
Reporting year	*n*	%	*n*	%	*n*	%	*n*	%	*n*	%	*n*	%	*n*	%
2005	150	1.82	150	3.80										
2006	570	6.92	570	14.44										
2007	899	10.91	899	22.77										
2008	471	5.72	471	11.93										
2009	188	2.28	188	4.76										
2010	178	2.16	98	2.48	80	6.94								
2011	188	2.28	56	1.42	132	11.46								
2012	86	1.04	48	1.22	38	3.30								
2013	102	1.24	59	1.49	43	3.73								
2014	91	1.10	68	1.72	23	2.00								
2015	333	4.04	191	4.84	97	8.42			45	2.45				
2016	399	4.84	141	3.57	131	11.37			127	6.93				
2017	449	5.45	186	4.71	76	6.60	2	16.67	185	10.09				
2018	596	7.23	212	5.37	133	11.55	2	16.67	223	12.17	26	2.52		
2019	569	6.91	183	4.64	66	5.73	1	8.33	264	14.40	55	5.32		
2020	732	8.88	181	4.58	93	8.07	1	8.33	306	16.69	151	14.62		
2021	935	11.35	146	3.70	129	11.20	0	0.00	431	23.51	229	22.17		
2022	893	10.84	81	2.05	75	6.51	6	50.00	191	10.42	382	36.98	158	60.31
Q1 2023	411	4.99	20	0.51	36	3.13	0	0.00	61	3.33	190	18.39	104	39.69
Occupation of reporters	*n*	%	*n*	%	*n*	%	*n*	%	*n*	%	*n*	%	*n*	%
Consumer	6,577	79.82	3,067	77.68	829	71.96	12	100.00	1,647	89.85	776	75.12	246	93.89
Physician	694	8.42	319	8.08	169	14.67	0	0.00	70	3.82	128	12.39	8	3.05
Pharmacist	137	1.66	38	0.96	31	2.69	0	0.00	35	1.91	33	3.19	0	0.00
Health professional	161	1.95	8	0.20	25	2.17	0	0.00	43	2.35	78	7.55	7	2.67
Other health professional	202	2.45	86	2.18	75	6.51	0	0.00	28	1.53	13	1.26	0	0.00
Lawyer	12	0.15	6	0.15	3	0.26	0	0.00	1	0.05	1	0.10	1	0.38
Unknown	457	5.55	424	10.74	20	1.74	0	0.00	9	0.49	4	0.39	0	0.00
Indications	*n*	%	*n*	%	*n*	%	*n*	%	*n*	%	*n*	%	*n*	%
Diabetes mellitus	5,286	64.15	3,287	83.26	582	50.52	7	58.33	949	51.77	385	37.27	76	29.01
Weight control	332	4.03	32	0.81	160	13.89	0	0.00	11	0.60	93	9.00	36	13.74
Blood glucose abnormal	78	0.95	46	1.17	6	0.52	0	0.00	15	0.82	9	0.87	2	0.76
Others	263	3.19	136	3.44	27	2.34	0	0.00	28	1.53	42	4.07	30	11.45
Unknown	2,281	27.68	447	11.32	377	32.73	5	41.67	830	45.28	504	48.79	118	45.04
Outcomes	*n*	%	*n*	%	*n*	%	*n*	%	*n*	%	*n*	%	*n*	%
Death	79	0.96	47	1.19	15	1.30	0	0.00	11	0.60	6	0.58	0	0.00
Life threatening	43	0.52	16	0.41	12	1.04	0	0.00	4	0.22	10	0.97	1	0.38
Hospitalization	883	10.72	489	12.39	124	10.76	1	8.33	168	9.17	87	8.42	14	5.34
Disability	111	1.35	39	0.99	25	2.17	0	0.00	22	1.20	23	2.23	2	0.76
Congenital anomaly	1	0.01	1	0.03	0	0.00	0	0.00	0	0.00	0	0.00	0	0.00
Required intervention to prevent	14	0.17	8	0.20	2	0.17	0	0.00	1	0.05	3	0.29	0	0.00
Other medical event	1,064	12.91	341	8.64	209	18.14	4	33.33	262	14.29	232	22.46	16	6.11
Unknown	6,045	73.36	3,007	76.17	765	66.41	7	58.33	1,365	74.47	672	65.05	229	87.40
The time to onset (days)	*n*	%	*n*	%	*n*	%	*n*	%	*n*	%	*n*	%	*n*	%
0–30	734		403	39.05	100	64.52	1		111		92	66.19	27	67.50
30–60	206		152	14.73	15	9.68			14		19	13.67	6	15.00
60–90	102		81	7.85	6	3.87			3		10	7.19	2	5.00
90–120	57		45	4.36	5	3.23			1		4	2.88	2	5.00
120–150	42		35	3.39	1	0.65			2		2	1.44	2	5.00
150–180	29		22	2.13	3	1.94			2		2	1.44	0	0.00
180–360	118		100	9.69	10	6.45			5		2	1.44	1	2.50
360+	223		194	18.80	15	9.68			6		8	5.76	0	0.00
Median (IQR)	31 (7–145.4)		45 (11–212.5)		16 (5.5–61)				7 (2–9)		12 (4–45.5)		10 (2–35.5)	

### Time-to-onset analysis

**Figure 2:**
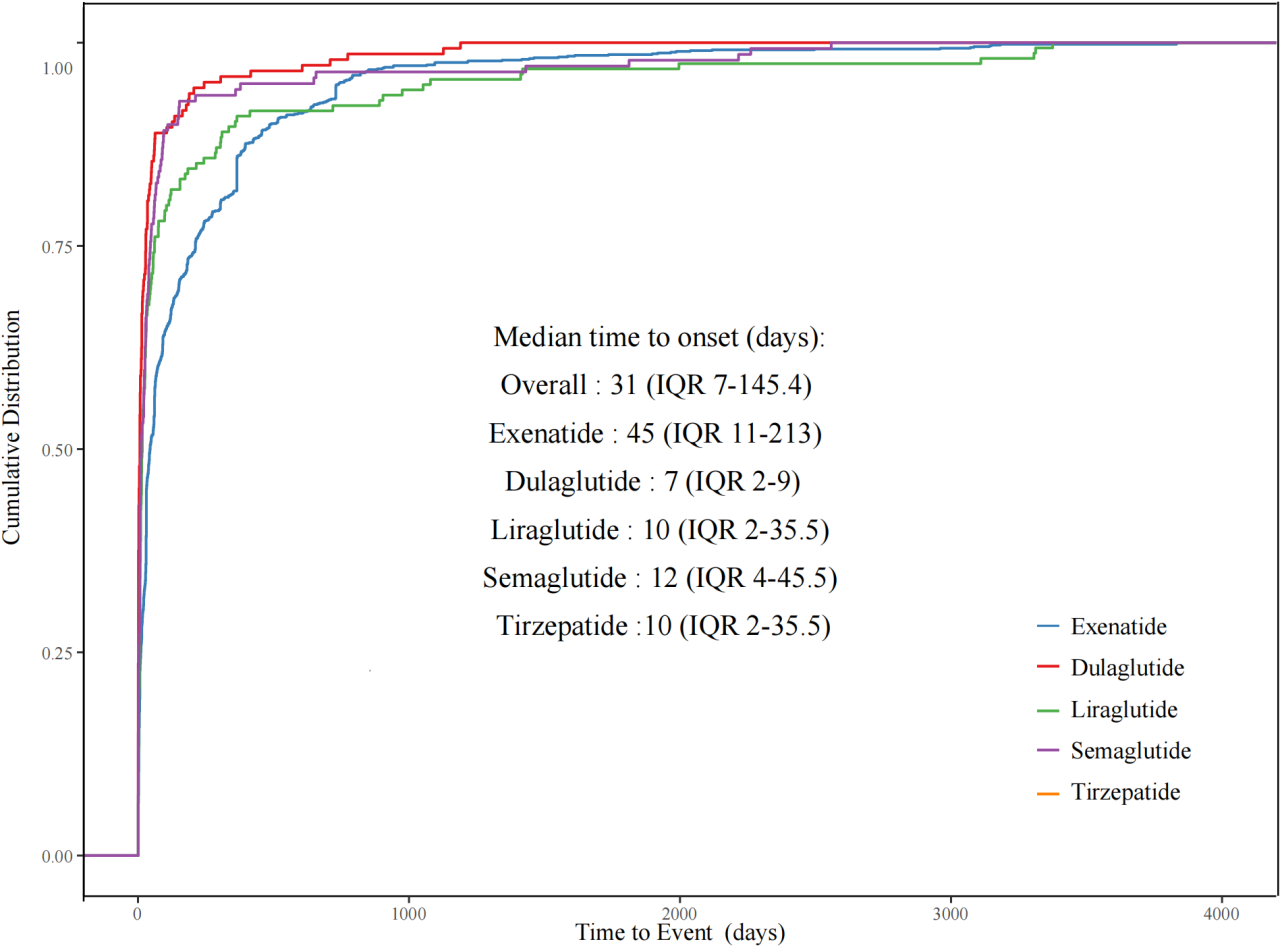
Cumulative distribution curves demonstrating the onset time of glucagon-like peptide-1 receptor agonist (GLP-1 RA)-related psychiatric adverse events (pAEs) after treatment with different GLP-1 RAs.

Of all the validated reports, the median time to onset of psychiatric AEs for the overall GLP-1 RAs was 31 days (IQR = 7–145.4 days), with 48.58% of the AEs occurring within the initial 30 days. Among individual GLP-1 RAs, exenatide exhibited the longest median time to onset at 45 days (IQR = 11–213 days), while dulaglutide had the shortest at 7 days (IQR = 2–9 days), liraglutide at 16 days (IQR = 5.5–61 days), semaglutide at 12 days (IQR = 4–45.5 days), and tirzepatide at 10 days (IQR = 2–35.5 days). Since lixisenatide lacks sufficient effective onset time, the time-to-onset statistics were not done. We made a comparison of the time-to-onset data available for the different GLP-1 RA agents. As shown in **Figure 2**, the cumulative distribution curves indicate the time to onset of psychiatric AEs after treatment with different GLP-1 RAs, with a significant difference for exenatide compared with the other four GLP-1 RAs (*p*< 0.001).

### Scanning for GLP-1 RA-related psychiatric AEs

Signals of psychiatric AEs reported in GLP-1 RA treatments were categorized by PT level (**Supplementary Table S2**). We characterized the classes and the number of reports of psychiatric AEs caused by GLP-1 RAs. The top six groups of psychiatric AEs with the highest number of reports were insomnia (*n* = 1,198, 11.70%), anxiety (*n* = 1,131, 11.04%), nervousness (*n* = 941, 9.19%), depression (*n* = 770, 7.52%), stress (*n* = 770, 7.52%), and confusional state (*n* = 689, 6.73%).

**Figure 3:**
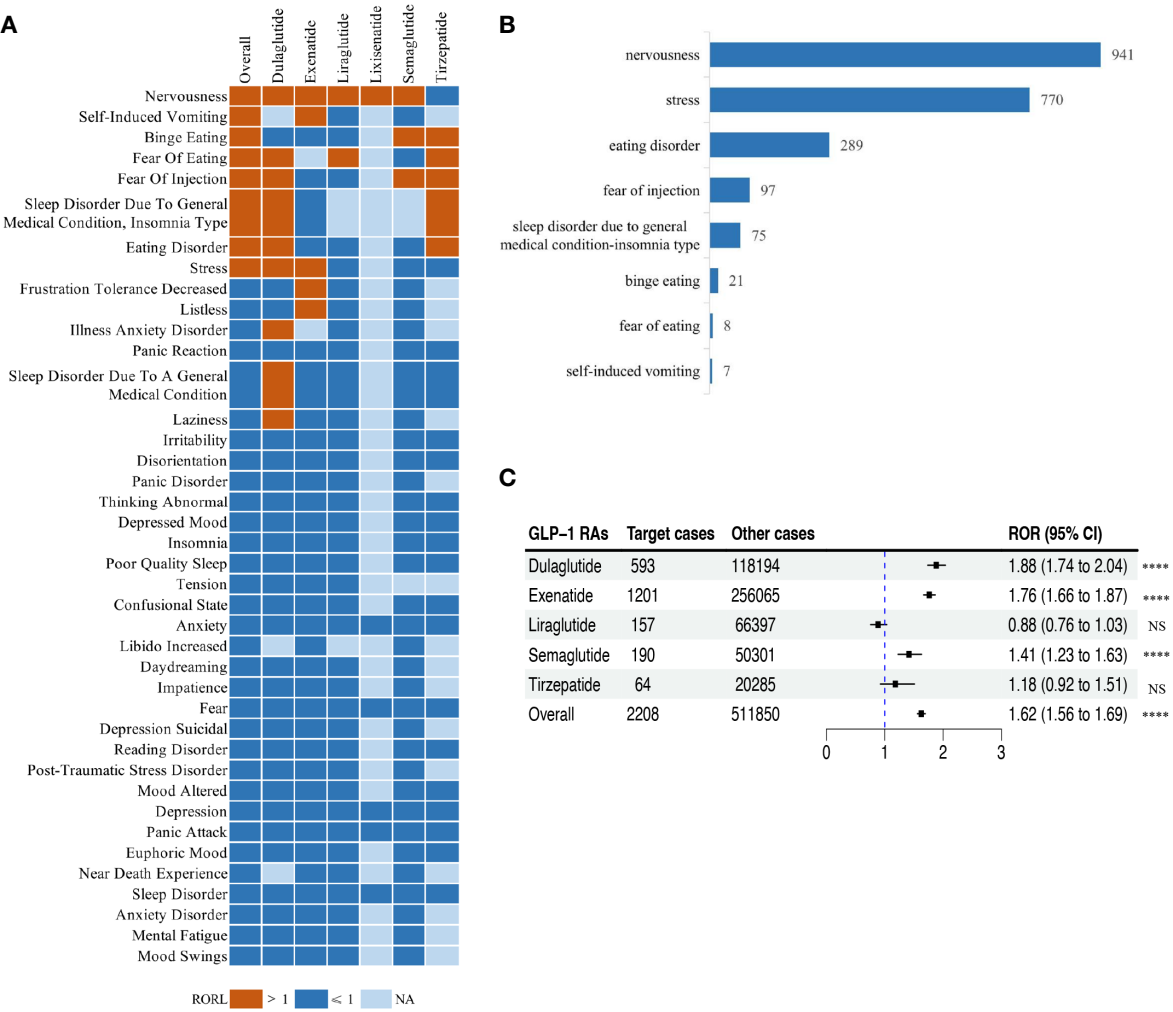
Scanning for glucagon-like peptide-1 receptor agonist (GLP-1 RA)-related psychiatric adverse events (AEs) based on the FDA Adverse Event Reporting System (FAERS) database. **(A)** Heatmap showing the reporting odds ratio (ROR) for the top 40 psychiatric AEs (with reports no less than 5) in the FAERS database under different GLP-1 RA treatment strategies (including overall, exenatide, liraglutide, lixisenatide, dulaglutide, semaglutide, and tirzepatide). *Red* indicates the lower limit of the 95% confidence interval for an ROR greater than 1, *dark blue* indicates the lower limit of the 95% confidence interval for an ROR less than 1, and *light blue* indicates ROR values that could not be calculated. **(B)** Bar plot showing the number of reported cases for eight categories of GLP-1 RA-related psychiatric AEs with different GLP-1 RA treatments. **(C)** Forest plot showing the ROR of GLP-1 RA-related psychiatric AEs with different GLP-1 RA treatments. *NS*, not significant. ****p< 0.0001.

We calculated the ROR of the PTs (*n* = 92) with no less than 5 reports of psychiatric AEs among treatments with GLP-1 RAs, using the entire FAERS database as a benchmark, and then performed a disproportionality analysis (**Supplementary Figure S1**). After screening according to the criteria for positive signals, we identified different psychiatric AEs related to different GPL-1 RA therapies. **Figure 3A** illustrates the ROR for the top 40 psychiatric AEs in the FAERS database under various GLP-1 RA treatments. Ultimately, based on the full GLP-1 RA psychiatric AE reporting profile, we filtered eight positive signals for psychiatric AEs with ROR05 greater than 1 and defined them as GLP-1 RA-related psychiatric AEs, namely, nervousness (*n* = 941; ROR = 1.97, 95% CI = 1.85–2.11), stress (*n* = 770; ROR = 1.28, 95% CI = 1.19–1.38), eating disorder (*n* = 289; ROR = 1.57, 95% CI = 1.40–1.77), fear of injection (*n* = 97; ROR = 1.96, 95% CI = 1.60–2.40), sleep disorder due to general medical condition—insomnia type (*n* = 75; ROR = 2.01, 95% CI = 1.60–2.52), binge eating (*n* = 21; ROR = 2.70, 95% CI = 1.75–4.16), fear of eating (*n* = 8; ROR = 3.35, 95% CI = 1.65–6.78), and self-induced vomiting (*n* = 7; ROR = 3.77, 95% CI = 1.77–8.03), which were highly associated with treatment with GLP-1 RAs. Compared with the other positive signals, self-induced vomiting was one of the GLP-1 RA-related psychiatric AEs that had a higher ROR signal, but the lowest number of reports (**Figure 3B**).

For these eight GLP-1 RA-related psychiatric AE signals, we further calculated their ROR considering the eight types of GLP-1 RA-related psychiatric AEs as one category of AEs in the full FAERS database. In general, there was a significant association between treatment with GLP-1 RAs and the occurrence of GLP-1 RA-related psychiatric AEs (ROR = 1.62, 95% CI = 1.56–1.69), which varied with respect to the different GLP-1 RAs (**Figure 3C**). In addition, treatments with liraglutide, semaglutide, or tirzepatide were significantly associated with the occurrence of GLP-1 RA-related psychiatric AEs with relatively low ROR (ROR_liraglutide_ = 0.88, 95% CI = 0.76–1.03, *p* = 0.0993; ROR_semaglutide_ = 1.41, 95% CI = 1.23–1.63, *p*< 0.001; ROR_tirzepatide_ = 1.18, 95% CI = 0.92–1.51, *p* = 0.1904).

## Discussion

GLP-1 RAs hold a prominent position in the diabetes and obesity treatment paradigm due to their multiple advantages, such as robust glycemic control, relatively low risk of hypoglycemia, favorable adherence, and cardiovascular benefits. Moreover, long-acting GLP-1 RAs have been approved for the treatment of obesity due to their improved tolerability, safety, and efficacy (19). As described in *Introduction*, previous investigations into GLP-1 RA-related AEs primarily concentrated on gastrointestinal- and tumor-related issues. However, there remains a dearth of comprehensive studies specifically exploring potential psychiatric AEs associated with GLP-1 RA therapy. Our study represents the first pharmacovigilance investigation into the potential risk of psychiatric AEs associated with GLP-1 RAs that utilizes real-world data extracted from the FAERS database. We examined reports of GLP-1 RA-induced psychiatric AEs using the entire FAERS database as a comparator, detailing the real-world reporting of such AEs and the corresponding clinical characteristics. By means of disproportionate analysis methods, we identified eight psychiatric AEs highly associated with GLP-1 RA treatment and analyzed the data on their variability.

Early clinical trials of GLP-1 RAs did not demonstrate a correlation between GLP-1 RA utilization and psychiatric AE occurrence (10). The results of a randomized controlled trial of liraglutide for weight management showed no clinically relevant between-group differences in mental health assessments, including psychiatric AEs and questionnaire-based scores assessing suicidal ideation or behavior (20). The results of another study on weight loss in people without diabetes with semaglutide and liraglutide treatment showed that more participants reported psychiatric-related AEs induced by liraglutide compared with either semaglutide or placebo, largely due to differences in the insomnia events [semaglutide: *n* = 3 (2.4%); liraglutide: *n* = 7 (5.5%); placebo: *n* = 2 (2.4%)] (21). To date, only one case of possible depression induced by exenatide has been reported (22). Given that both diabetes and obesity confer high psychiatric disorder risks, an increasing number of researchers have begun investigating a potential relationship between the use of GLP-1 RAs and the occurrence of psychiatric AEs (23–25).

Psychiatric AEs are a less frequent class of adverse reactions associated with GLP-1 RAs. Analysis of the FAERS database showed that psychiatric AEs occurred in 4.55% of the reported adverse reactions in patients treated with GLP-1 RAs during the period 2004–2023. The proportion of psychiatric AEs reported as adverse reactions in patients treated with semaglutide (5.82%) was slightly higher than that in patients treated with other GLP-1 RAs. The lowest proportion of psychiatric AEs was observed with tirzepatide at 2.71%. The shorter time since market approval could explain the fewer psychiatric AEs. Semaglutide had been approved for both diabetes and obesity treatment, while tirzepatide was only approved for obesity in November of 2023 (26, 27). The more recent approval and availability of tirzepatide has allowed less time for AEs to be reported into the FAERS database compared to semaglutide, in particular the obesity indication. Notably, we found that a significantly higher proportion of psychiatric adverse reactions to GLP-1 RAs were reported in women compared to men. Interestingly, according to the International Diabetes Federation (IDF) assessment, the prevalence of diabetes is currently slightly higher in men than in women. This phenomenon could potentially be attributed to the fact that 79.82% of our reports were submitted by consumers on a voluntary basis, thus suggesting a plausible inclination for women to report AEs to FAERS as opposed to men (28). Further studies are needed on the gender differences in psychiatric AEs caused by treatment with GLP-1 RAs. In addition, the psychiatric AEs from GLP-1 RA treatment are primarily from self-reporting by consumers, and the increase in the number of AEs could be attributed to the expansion of the indications for GLP-1 RAs and the increased awareness of psychiatric AEs among healthcare professionals and patients during post-market surveillance.

Regarding the time of onset, our study showed that the median time to onset of the overall GLP-1 RA AEs was 31 days (IQR = 7–145.4 days). Of note is that the observed median time to onset of the psychiatric AEs in our analysis of the FAERS data aligned with common gastrointestinal AEs such as nausea and vomiting, which were the most frequently reported within the first 30 days of GLP-1 RA treatment (12). It can be inferred that frequent digestive reactions such as persistent nausea and vomiting are extremely likely to cause patients to experience emotional and sleep psychiatric problems. This may partially explain the association we observed between GLP-1 RAs and specific psychiatric adverse reactions. Future research still needs to further verify this potential mechanism connection. Overall, physicians and patients should pay attention to the potential psychiatric adverse reactions caused by treatment with GLP-1 RAs and to closely monitor them during the initial treatment. Among these GLP-1 RAs, exenatide had a significantly longer onset time, with statistically significant differences from the onset times of the other four GLP-1 RAs. GLP-1 RAs differ in their pharmacokinetic and pharmacodynamic properties, and recently developed agonists have a longer duration of action than endogenous GLP-1 RAs and the first synthetic GLP-1 RA, exenatide (29). These ligands also differ in their ability to activate the second messenger system and the degree to which they stimulate receptor internalization. GLP-1 is involved in the regulation of neural circuits, which play an important role in the regulation of mood, cognition, and behavior, and abnormalities in neural circuits could contribute to the development of psychiatric AEs (11). The exact mechanisms of action are unclear, and additional studies are needed to elucidate the underlying factors associated with the regimen-specific time to onset. The results of the time-to-onset study suggest that monitoring for psychiatric AEs can be appropriately prolonged for the use of exenatide compared with other GLP-1 RAs.

In this extensive pharmacovigilance study, we observed a significant association between GLP-1 RAs and eight distinct categories of psychiatric AEs, namely, nervousness, stress, eating disorder, fear of injection, sleep disorder due to general medical condition—insomnia type, binge eating, fear of eating, and self-induced vomiting. This study screened reports of adverse reactions to GLP-1 RAs with the aim of demonstrating that GLP-1 RA-associated psychiatric AEs are highly correlated with GLP-1 RA treatment. One of the plausible explanations for the association between GLP-1 RAs and the aforementioned psychiatric AEs might be the preexisting or latent psychiatric disorder in GLP-1 RA users. According to the literature, the prevalence of depression is significantly higher in patients with type 2 diabetes than in those without diabetes (17.6% *vs*. 9.8%, OR = 1.6, 95% CI = 1.2–2.0) (30), and psychiatric AEs, especially eating disorders (binge eating and self-induced vomiting), are frequently observed in patients with diabetes and obesity (31, 32). Although there is no definitive evidence regarding the causal relationship between the use of GLP-1 RAs in humans and the development of psychiatric AEs, the fact that psychiatric AEs have occurred after the use of GLP-1 RAs suggests that these reports warrant increased clinical attention, early detection, and intervention. Epidemiologic reviews have displayed a bidirectional relationship between diabetes and depression, suggesting that patients with depression potentially have a 41% increased risk of developing diabetes, while patients with type 2 diabetes would have a twofold risk of depression as controls (33). A portion of the GLP-1 receptors in neurons are located in the nucleus of the solitary tract (NTS), which is functionally connected to the hypothalamus and hindbrain, and the GLP-1 generated in the NTS works by binding to the receptors in the plasma membrane of the neurons directly involved in appetite control (34). On the other hand, the use of GLP-1 RAs has been shown to be correlated with emotional eating and depression in patients with diabetes and obesity (35). Although there are only limited animal experiments suggesting that GLP-1 may lead to the development of psychiatric AEs (11), and there is no definitive evidence regarding the causal relationship between the use of GLP-1 RAs in humans and the development of psychiatric AEs, the fact that psychiatric AEs have occurred after the use of GLP-1 RAs suggests that these reports warrant increased clinical attention, early detection, and intervention.

Importantly, our study assessed and compared the signal strength of the GLP-1 RA-related psychiatric AEs across different GLP-1 RA therapies. Significant differences in the reporting odds ratios of the GLP-1 RA-related psychiatric AEs were observed. Liraglutide (ROR = 0.88, 95% CI = 0.76–1.03) demonstrated a significantly lower risk of psychiatric AEs compared to the overall GLP-1 RAs (ROR = 1.62, 95% CI = 1.56–1.69). In addition, semaglutide (ROR = 1.41, 95% CI = 1.23–1.63) exhibited a reduced GLP-1 RA-associated psychiatric AE risk compared to dulaglutide (ROR = 1.88, 95% CI = 1.74–2.04). Conclusions regarding the influencing factors of GLP-1 RA-related psychiatric AEs require further validation through larger studies or clinical trials.

This study has several limitations. Firstly, the FAERS database functions as a global spontaneous reporting system and inherently carries limitations such as uncertainty regarding the causality between the reported events and products, potential incomplete reporting, inaccuracies due to the public’s level of awareness of a particular adverse reaction, and substantial missing information (e.g., medication dates, age, and weight, among others). Furthermore, FAERS includes only cases where AEs occurred rather than complete adverse reaction reports. Given the absence of a baseline prevalence for these psychiatric conditions, it becomes more challenging to ascertain whether they were preexisting or potential AEs, particularly considering that 79.82% of the cases were reported by consumers. Furthermore, due to the absence of expertise in individual reporting, the extent of the correlation between the reported AEs and the product remains inconclusive. We were unable to obtain a causal relationship between GLP-1 RAs and psychiatric AEs or to calculate the incidence of psychiatric AEs caused by GLP-1 RAs. In addition, we did not provide robust evidence on the pharmacological mechanisms of the GLP-1 RA-related psychiatric AEs. The identified GLP-1 RA-related psychiatric AEs require clinical validation because of the minimal existing research on this topic. FAERS-based disproportionality analyses neither showed causality nor quantified risk, merely statistical association strengths; thus, our findings on GLP-1-related psychiatric AEs need to be confirmed by large prospective studies.

## Conclusion

Through a pharmacovigilance study, we conducted an exhaustive and systematic extraction and analysis of reports detailing psychiatric AEs linked to the administration of GLP-1 RAs. Eight categories of psychiatric AEs highly associated with GLP-1 RAs were comprehensively investigated and identified by performing a disproportionality analysis. Although several limitations exist in the analysis of the FAERS database, the preliminary findings in this work could provide a better basis for understanding the potential psychiatric AEs that may occur with GLP-1 RA treatment, assisting clinicians to focus on these AEs and provide early intervene for optimal risk management.

## Data availability statement

The original contributions presented in the study are included in the article/**Supplementary Material**. Further inquiries can be directed to the corresponding authors.

## Author contributions

WC: Conceptualization, Methodology, Validation, Writing – original draft, Writing – review & editing. PC: Formal analysis, Investigation, Methodology, Writing – original draft. WZ: Formal analysis, Software, Supervision, Writing – review & editing. ZF: Conceptualization, Funding acquisition, Supervision, Writing – review & editing, Writing – original draft.
