# Supplementary material for: Psychiatric adverse events associated with GLP-1 receptor agonists: a real-world pharmacovigilance study based on the FDA Adverse Event Reporting System database
Source: Front Endocrinol (Lausanne). 2024 Feb 6;15:1330936. doi: 10.3389/fendo.2024.1330936 (PMC10882716; doi:10.3389/fendo.2024.1330936)
Supplement: Supplementary file 1 [file DataSheet_1.docx]

Figure S1. The heatmap shows the ROR for all detected psychiatric adverse events (with reports no less than 5) in the FAERS database under different GLP-1 RAs treatment strategies.


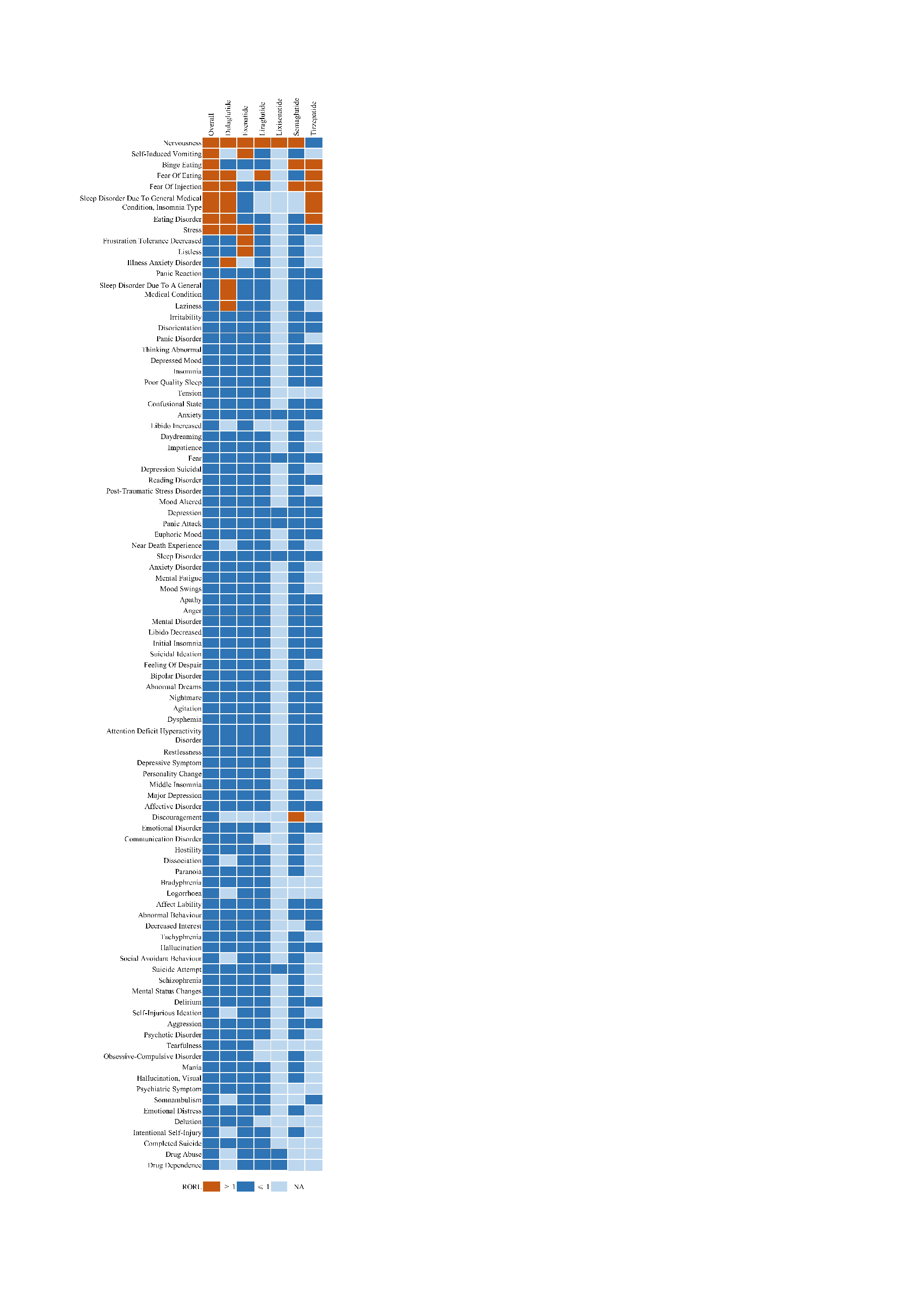


**Table S1. Product information for different Glucagon-like peptide-1 receptor agonists (GLP-1 RAs)**

| GLP-1 RAs | Original Research Company | Trade Name | Marketing Date | Indications |
| --- | --- | --- | --- | --- |
| Exenatide | Eli Lilly & Co. Amylin Pharmaceuticals, Inc. | BYETTA, BYDUREON | 2005.04 | Type 2 diabetes mellitus |
| Liraglutide | Novo Nordisk A/S | VICTOZA,  SAXENDA | 2009.06 | Cardiovascular disease,  Obesity,  Overweight  Type 2 diabetes mellitus |
| Lixisenatide | Zealand Pharma A/S | ADLYXIN | 2013.01 | Type 2 diabetes mellitus |
| Dulaglutide | Eli Lilly & Co. | TRULICITY | 2014.09 | Type 2 diabetes mellitus |
| Lixisenatide | Sanofi | SOLIQUA | 2016.11 | Type 2 diabetes mellitus |
| Semaglutide | Novo Nordisk A/S | OZEMPIC,  RYBELSUS,  WEGOVY | 2017.12 | Overweight,  Type 2 diabetes mellitus |
| Tirzepatide | Eli Lilly & Co. | MOUNJARO | 2022.05 | Type 2 diabetes mellitus,  Obesity,  Overweight |

| **Table S2. The case number of different psychiatric adverse events in cases receiving Glucagon-like peptide-1 receptor agonists (GLP-1 RAs) treatment in FAERS database from 2004-2023（n=10,242）** | | | | | | |
| --- | --- | --- | --- | --- | --- | --- |
| pt_name_en | numbers of  aim AEs | percent | ROR | RORL | RORU | ROR 95%Cl |
| Insomnia | 1198 | 11.70% | 0.5157 | 0.4872 | 0.5458 | 0.52(0.49-0.55) |
| Anxiety | 1131 | 11.04% | 0.4499 | 0.4244 | 0.4770 | 0.45(0.42-0.48) |
| Nervousness | 941 | 9.19% | 1.9742 | 1.8507 | 2.1059 | 1.97(1.85-2.11) |
| Depression | 770 | 7.52% | 0.3745 | 0.3489 | 0.4020 | 0.37(0.35-0.40) |
| Stress | 770 | 7.52% | 1.2809 | 1.1929 | 1.3754 | 1.28(1.19-1.38) |
| Confusional state | 689 | 6.73% | 0.4920 | 0.4565 | 0.5303 | 0.49(0.46-0.53) |
| Irritability | 361 | 3.52% | 0.6735 | 0.6073 | 0.7470 | 0.67(0.61-0.75) |
| Eating disorder | 289 | 2.82% | 1.5730 | 1.4003 | 1.7669 | 1.57(1.40-1.77) |
| Depressed mood | 268 | 2.62% | 0.6113 | 0.5421 | 0.6893 | 0.61(0.54-0.69) |
| Disorientation | 242 | 2.36% | 0.6783 | 0.5978 | 0.7698 | 0.68(0.60-0.77) |
| Suicidal ideation | 197 | 1.92% | 0.2491 | 0.2166 | 0.2865 | 0.25(0.22-0.29) |
| Sleep disorder | 184 | 1.80% | 0.3324 | 0.2876 | 0.3842 | 0.33(0.29-0.38) |
| Agitation | 152 | 1.48% | 0.2291 | 0.1954 | 0.2686 | 0.23(0.20-0.27) |
| Panic attack | 121 | 1.18% | 0.3859 | 0.3228 | 0.4613 | 0.39(0.32-0.46) |
| Fear | 120 | 1.17% | 0.4695 | 0.3924 | 0.5618 | 0.47(0.39-0.56) |
| Thinking abnormal | 117 | 1.14% | 0.6891 | 0.5745 | 0.8265 | 0.69(0.57-0.83) |
| Frustration tolerance decreased | 115 | 1.12% | 1.1457 | 0.9532 | 1.3769 | 1.15(0.95-1.38) |
| Mental disorder | 111 | 1.08% | 0.2979 | 0.2472 | 0.3589 | 0.30(0.25-0.36) |
| Poor quality sleep | 103 | 1.01% | 0.5887 | 0.4850 | 0.7146 | 0.59(0.49-0.71) |
| Mood altered | 102 | 1.00% | 0.4412 | 0.3632 | 0.5360 | 0.44(0.36-0.54) |
| Fear of injection | 97 | 0.95% | 1.9621 | 1.6047 | 2.3990 | 1.96(1.60-2.40) |
| Mood swings | 94 | 0.92% | 0.3259 | 0.2661 | 0.3990 | 0.33(0.27-0.40) |
| Anger | 93 | 0.91% | 0.3037 | 0.2478 | 0.3723 | 0.30(0.25-0.37) |
| Restlessness | 77 | 0.75% | 0.2390 | 0.1911 | 0.2989 | 0.24(0.19-0.30) |
| Hallucination | 76 | 0.74% | 0.1253 | 0.1001 | 0.1569 | 0.13(0.10-0.16) |
| Nightmare | 76 | 0.74% | 0.2485 | 0.1984 | 0.3113 | 0.25(0.20-0.31) |
| Sleep disorder due to general medical condition, insomnia type | 75 | 0.73% | 2.0054 | 1.5955 | 2.5208 | 2.01(1.60-2.52) |
| Sleep disorder due to a general medical condition | 73 | 0.71% | 0.8761 | 0.6958 | 1.1032 | 0.88(0.70-1.10) |
| Abnormal dreams | 66 | 0.64% | 0.2570 | 0.2019 | 0.3273 | 0.26(0.20-0.33) |
| Panic reaction | 61 | 0.60% | 0.9665 | 0.7510 | 1.2437 | 0.97(0.75-1.24) |
| Abnormal behaviour | 57 | 0.56% | 0.1526 | 0.1177 | 0.1979 | 0.15(0.12-0.20) |
| Suicide attempt | 56 | 0.55% | 0.1081 | 0.0832 | 0.1405 | 0.11(0.08-0.14) |
| Emotional disorder | 54 | 0.53% | 0.1930 | 0.1478 | 0.2521 | 0.19(0.15-0.25) |
| Emotional distress | 49 | 0.48% | 0.0412 | 0.0312 | 0.0546 | 0.04(0.03-0.05) |
| Aggression | 44 | 0.43% | 0.0977 | 0.0727 | 0.1313 | 0.10(0.07-0.13) |
| Apathy | 42 | 0.41% | 0.3418 | 0.2524 | 0.4627 | 0.34(0.25-0.46) |
| Euphoric mood | 41 | 0.40% | 0.4216 | 0.3102 | 0.5730 | 0.42(0.31-0.57) |
| Listless | 38 | 0.37% | 1.2891 | 0.9360 | 1.7754 | 1.29(0.94-1.78) |
| Middle insomnia | 37 | 0.36% | 0.2417 | 0.1750 | 0.3337 | 0.24(0.18-0.33) |
| Tension | 36 | 0.35% | 0.6615 | 0.4766 | 0.9181 | 0.66(0.48-0.92) |
| Libido decreased | 35 | 0.34% | 0.3228 | 0.2316 | 0.4498 | 0.32(0.23-0.45) |
| Delirium | 31 | 0.30% | 0.1106 | 0.0778 | 0.1573 | 0.11(0.08-0.16) |
| Panic disorder | 30 | 0.29% | 0.8447 | 0.5897 | 1.2101 | 0.84(0.59-1.21) |
| Bipolar disorder | 29 | 0.28% | 0.2915 | 0.2025 | 0.4198 | 0.29(0.20-0.42) |
| Post-traumatic stress disorder | 29 | 0.28% | 0.5331 | 0.3701 | 0.7679 | 0.53(0.37-0.77) |
| Mental status changes | 28 | 0.27% | 0.1147 | 0.0792 | 0.1661 | 0.11(0.08-0.17) |
| Paranoia | 28 | 0.27% | 0.1828 | 0.1261 | 0.2648 | 0.18(0.13-0.26) |
| Psychotic disorder | 27 | 0.26% | 0.1050 | 0.0720 | 0.1532 | 0.11(0.07-0.15) |
| Initial insomnia | 27 | 0.26% | 0.3358 | 0.2301 | 0.4900 | 0.34(0.23-0.49) |
| Personality change | 24 | 0.23% | 0.2776 | 0.1860 | 0.4144 | 0.28(0.19-0.41) |
| Binge eating | 21 | 0.21% | 2.6972 | 1.7480 | 4.1617 | 2.70(1.75-4.16) |
| Depression suicidal | 18 | 0.18% | 0.6037 | 0.3798 | 0.9597 | 0.60(0.38-0.96) |
| Affect lability | 17 | 0.17% | 0.1920 | 0.1193 | 0.3091 | 0.19(0.12-0.31) |
| Affective disorder | 17 | 0.17% | 0.2645 | 0.1643 | 0.4258 | 0.26(0.16-0.43) |
| Major depression | 17 | 0.17% | 0.2817 | 0.1750 | 0.4534 | 0.28(0.17-0.45) |
| Attention deficit hyperactivity disorder | 16 | 0.16% | 0.3182 | 0.1948 | 0.5198 | 0.32(0.19-0.52) |
| Completed suicide | 15 | 0.15% | 0.0197 | 0.0119 | 0.0326 | 0.02(0.01-0.03) |
| Hallucination, visual | 15 | 0.15% | 0.0885 | 0.0533 | 0.1468 | 0.09(0.05-0.15) |
| Laziness | 15 | 0.15% | 1.0556 | 0.6346 | 1.7559 | 1.06(0.63-1.76) |
| Mania | 14 | 0.14% | 0.0971 | 0.0575 | 0.1640 | 0.10(0.06-0.16) |
| Schizophrenia | 14 | 0.14% | 0.1354 | 0.0802 | 0.2288 | 0.14(0.08-0.23) |
| Feeling of despair | 14 | 0.14% | 0.3639 | 0.2153 | 0.6151 | 0.36(0.22-0.62) |
| Anxiety disorder | 14 | 0.14% | 0.4697 | 0.2778 | 0.7942 | 0.47(0.28-0.79) |
| Bradyphrenia | 13 | 0.13% | 0.2165 | 0.1256 | 0.3731 | 0.22(0.13-0.37) |
| Dysphemia | 13 | 0.13% | 0.3363 | 0.1951 | 0.5797 | 0.34(0.20-0.58) |
| Reading disorder | 13 | 0.13% | 0.6502 | 0.3768 | 1.1218 | 0.65(0.38-1.12) |
| Libido increased | 13 | 0.13% | 0.7001 | 0.4057 | 1.2081 | 0.70(0.41-1.21) |
| Impatience | 12 | 0.12% | 0.6927 | 0.3926 | 1.2223 | 0.69(0.39-1.22) |
| Decreased interest | 11 | 0.11% | 0.2067 | 0.1144 | 0.3734 | 0.21(0.11-0.37) |
| Near death experience | 11 | 0.11% | 0.5277 | 0.2918 | 0.9545 | 0.53(0.29-0.95) |
| Dissociation | 10 | 0.10% | 0.2471 | 0.1328 | 0.4596 | 0.25(0.13-0.46) |
| Depressive symptom | 10 | 0.10% | 0.3528 | 0.1896 | 0.6564 | 0.35(0.19-0.66) |
| Daydreaming | 10 | 0.10% | 0.7409 | 0.3977 | 1.3803 | 0.74(0.40-1.38) |
| Obsessive-compulsive disorder | 9 | 0.09% | 0.1208 | 0.0629 | 0.2324 | 0.12(0.06-0.23) |
| Social avoidant behaviour | 9 | 0.09% | 0.1623 | 0.0844 | 0.3122 | 0.16(0.08-0.31) |
| Communication disorder | 9 | 0.09% | 0.2711 | 0.1409 | 0.5215 | 0.27(0.14-0.52) |
| Drug dependence | 8 | 0.08% | 0.0049 | 0.0025 | 0.0099 | 0.00(0.00-0.01) |
| Drug abuse | 8 | 0.08% | 0.0111 | 0.0055 | 0.0222 | 0.01(0.01-0.02) |
| Intentional self-injury | 8 | 0.08% | 0.0381 | 0.0190 | 0.0762 | 0.04(0.02-0.08) |
| Delusion | 8 | 0.08% | 0.0622 | 0.0311 | 0.1245 | 0.06(0.03-0.12) |
| Logorrhoea | 8 | 0.08% | 0.2478 | 0.1238 | 0.4961 | 0.25(0.12-0.50) |
| Fear of eating | 8 | 0.08% | 3.3483 | 1.6544 | 6.7766 | 3.35(1.65-6.78) |
| Tachyphrenia | 7 | 0.07% | 0.2321 | 0.1105 | 0.4873 | 0.23(0.11-0.49) |
| Hostility | 7 | 0.07% | 0.2790 | 0.1329 | 0.5859 | 0.28(0.13-0.59) |
| Self-induced vomiting | 7 | 0.07% | 3.7739 | 1.7732 | 8.0321 | 3.77(1.77-8.03) |
| Somnambulism | 6 | 0.06% | 0.0921 | 0.0413 | 0.2050 | 0.09(0.04-0.21) |
| Psychiatric symptom | 6 | 0.06% | 0.0934 | 0.0419 | 0.2079 | 0.09(0.04-0.21) |
| Tearfulness | 6 | 0.06% | 0.1539 | 0.0691 | 0.3429 | 0.15(0.07-0.34) |
| Self-injurious ideation | 6 | 0.06% | 0.1712 | 0.0769 | 0.3814 | 0.17(0.08-0.38) |
| Discouragement | 6 | 0.06% | 0.3402 | 0.1526 | 0.7583 | 0.34(0.15-0.76) |
| Mental fatigue | 6 | 0.06% | 0.6072 | 0.2721 | 1.3549 | 0.61(0.27-1.35) |
| Illness anxiety disorder | 6 | 0.06% | 1.7781 | 0.7929 | 3.9875 | 1.78(0.79-3.99) |
| Hallucination, auditory | 5 | 0.05% | 0.0370 | 0.0154 | 0.0889 | 0.04(0.02-0.09) |
| Tic | 5 | 0.05% | 0.1025 | 0.0426 | 0.2464 | 0.10(0.04-0.25) |
| Enuresis | 5 | 0.05% | 0.1478 | 0.0615 | 0.3555 | 0.15(0.06-0.36) |
| Bipolar I disorder | 5 | 0.05% | 0.2472 | 0.1028 | 0.5946 | 0.25(0.10-0.59) |
| Obsessive thoughts | 5 | 0.05% | 0.3035 | 0.1262 | 0.7302 | 0.30(0.13-0.73) |
| Somatic symptom disorder | 5 | 0.05% | 0.3170 | 0.1318 | 0.7628 | 0.32(0.13-0.76) |
| Inappropriate affect | 5 | 0.05% | 0.3355 | 0.1394 | 0.8074 | 0.34(0.14-0.81) |
| Anhedonia | 4 | 0.04% | 0.0152 | 0.0057 | 0.0406 | 0.02(0.01-0.04) |
| Alcohol abuse | 4 | 0.04% | 0.1585 | 0.0594 | 0.4227 | 0.16(0.06-0.42) |
| Conversion disorder | 4 | 0.04% | 0.2302 | 0.0863 | 0.6141 | 0.23(0.09-0.61) |
| Derealisation | 4 | 0.04% | 0.2316 | 0.0868 | 0.6179 | 0.23(0.09-0.62) |
| Violence-related symptom | 4 | 0.04% | 0.2703 | 0.1013 | 0.7213 | 0.27(0.10-0.72) |
| Phobia | 4 | 0.04% | 0.3615 | 0.1354 | 0.9649 | 0.36(0.14-0.96) |
| Fear of falling | 4 | 0.04% | 0.6176 | 0.2311 | 1.6509 | 0.62(0.23-1.65) |
| Bulimia nervosa | 4 | 0.04% | 1.0018 | 0.3741 | 2.6831 | 1.00(0.37-2.68) |
| Autoscopy | 4 | 0.04% | 1.0937 | 0.4082 | 2.9305 | 1.09(0.41-2.93) |
| Time perception altered | 4 | 0.04% | 1.3536 | 0.5045 | 3.6316 | 1.35(0.50-3.63) |
| Aversion | 4 | 0.04% | 1.7671 | 0.6573 | 4.7512 | 1.77(0.66-4.75) |
| Loss of libido | 3 | 0.03% | 0.0416 | 0.0134 | 0.1289 | 0.04(0.01-0.13) |
| Learning disability | 3 | 0.03% | 0.0558 | 0.0180 | 0.1731 | 0.06(0.02-0.17) |
| Catatonia | 3 | 0.03% | 0.0717 | 0.0231 | 0.2223 | 0.07(0.02-0.22) |
| Homicidal ideation | 3 | 0.03% | 0.0762 | 0.0246 | 0.2364 | 0.08(0.02-0.24) |
| Suicidal behaviour | 3 | 0.03% | 0.0795 | 0.0256 | 0.2465 | 0.08(0.03-0.25) |
| Psychomotor retardation | 3 | 0.03% | 0.1262 | 0.0407 | 0.3917 | 0.13(0.04-0.39) |
| Anorgasmia | 3 | 0.03% | 0.1322 | 0.0426 | 0.4103 | 0.13(0.04-0.41) |
| Staring | 3 | 0.03% | 0.1386 | 0.0447 | 0.4300 | 0.14(0.04-0.43) |
| Flat affect | 3 | 0.03% | 0.1573 | 0.0507 | 0.4882 | 0.16(0.05-0.49) |
| Fear of death | 3 | 0.03% | 0.1596 | 0.0514 | 0.4952 | 0.16(0.05-0.50) |
| Sleep talking | 3 | 0.03% | 0.2080 | 0.0670 | 0.6459 | 0.21(0.07-0.65) |
| Terminal insomnia | 3 | 0.03% | 0.2354 | 0.0758 | 0.7310 | 0.24(0.08-0.73) |
| Learning disorder | 3 | 0.03% | 0.2520 | 0.0812 | 0.7825 | 0.25(0.08-0.78) |
| Libido disorder | 3 | 0.03% | 0.2658 | 0.0856 | 0.8255 | 0.27(0.09-0.83) |
| Claustrophobia | 3 | 0.03% | 0.3095 | 0.0996 | 0.9614 | 0.31(0.10-0.96) |
| Psychotic behaviour | 3 | 0.03% | 0.3333 | 0.1073 | 1.0354 | 0.33(0.11-1.04) |
| Neurosis | 3 | 0.03% | 0.7716 | 0.2477 | 2.4035 | 0.77(0.25-2.40) |
| Seasonal affective disorder | 3 | 0.03% | 1.0844 | 0.3475 | 3.3839 | 1.08(0.35-3.38) |
| Deja vu | 3 | 0.03% | 1.3315 | 0.4261 | 4.1612 | 1.33(0.43-4.16) |
| Bruxism | 2 | 0.02% | 0.0429 | 0.0107 | 0.1716 | 0.04(0.01-0.17) |
| Dysphoria | 2 | 0.02% | 0.0434 | 0.0109 | 0.1736 | 0.04(0.01-0.17) |
| Drug use disorder | 2 | 0.02% | 0.0473 | 0.0118 | 0.1892 | 0.05(0.01-0.19) |
| Autism spectrum disorder | 2 | 0.02% | 0.0540 | 0.0135 | 0.2158 | 0.05(0.01-0.22) |
| Hallucinations, mixed | 2 | 0.02% | 0.0593 | 0.0148 | 0.2372 | 0.06(0.01-0.24) |
| Sleep terror | 2 | 0.02% | 0.0685 | 0.0171 | 0.2742 | 0.07(0.02-0.27) |
| Negative thoughts | 2 | 0.02% | 0.0798 | 0.0199 | 0.3192 | 0.08(0.02-0.32) |
| Personality disorder | 2 | 0.02% | 0.0848 | 0.0212 | 0.3392 | 0.08(0.02-0.34) |
| Selective eating disorder | 2 | 0.02% | 0.0943 | 0.0236 | 0.3773 | 0.09(0.02-0.38) |
| Acute psychosis | 2 | 0.02% | 0.0950 | 0.0238 | 0.3803 | 0.10(0.02-0.38) |
| Fear of disease | 2 | 0.02% | 0.1137 | 0.0284 | 0.4551 | 0.11(0.03-0.46) |
| Psychotic symptom | 2 | 0.02% | 0.1374 | 0.0343 | 0.5499 | 0.14(0.03-0.55) |
| Emotional poverty | 2 | 0.02% | 0.1527 | 0.0381 | 0.6112 | 0.15(0.04-0.61) |
| Feelings of worthlessness | 2 | 0.02% | 0.1594 | 0.0398 | 0.6383 | 0.16(0.04-0.64) |
| Generalised anxiety disorder | 2 | 0.02% | 0.1657 | 0.0414 | 0.6632 | 0.17(0.04-0.66) |
| Disorganised speech | 2 | 0.02% | 0.1753 | 0.0438 | 0.7016 | 0.18(0.04-0.70) |
| Adjustment disorder | 2 | 0.02% | 0.1862 | 0.0465 | 0.7455 | 0.19(0.05-0.75) |
| Distractibility | 2 | 0.02% | 0.2009 | 0.0502 | 0.8045 | 0.20(0.05-0.80) |
| Abulia | 2 | 0.02% | 0.2647 | 0.0661 | 1.0605 | 0.26(0.07-1.06) |
| Orgasm abnormal | 2 | 0.02% | 0.2879 | 0.0718 | 1.1534 | 0.29(0.07-1.15) |
| Borderline personality disorder | 2 | 0.02% | 0.3331 | 0.0831 | 1.3350 | 0.33(0.08-1.34) |
| Thought blocking | 2 | 0.02% | 0.3809 | 0.0950 | 1.5274 | 0.38(0.10-1.53) |
| Flight of ideas | 2 | 0.02% | 0.3832 | 0.0956 | 1.5366 | 0.38(0.10-1.54) |
| Head banging | 2 | 0.02% | 0.3984 | 0.0994 | 1.5978 | 0.40(0.10-1.60) |
| Burnout syndrome | 2 | 0.02% | 0.4122 | 0.1028 | 1.6532 | 0.41(0.10-1.65) |
| Delusional perception | 2 | 0.02% | 0.5613 | 0.1398 | 2.2536 | 0.56(0.14-2.25) |
| Merycism | 2 | 0.02% | 0.8482 | 0.2108 | 3.4126 | 0.85(0.21-3.41) |
| Automatism | 2 | 0.02% | 0.9737 | 0.2418 | 3.9211 | 0.97(0.24-3.92) |
| Schizoaffective disorder bipolar type | 2 | 0.02% | 1.1928 | 0.2957 | 4.8109 | 1.19(0.30-4.81) |
| Verbigeration | 2 | 0.02% | 2.0973 | 0.5166 | 8.5140 | 2.10(0.52-8.51) |
| Purging | 2 | 0.02% | 2.7265 | 0.6686 | 11.1183 | 2.73(0.67-11.12) |
| Sleep disorder due to general medical condition, hypersomnia type | 2 | 0.02% | 5.1582 | 1.2432 | 21.4018 | 5.16(1.24-21.40) |
| Psychological trauma | 1 | 0.01% | 0.0114 | 0.0016 | 0.0809 | 0.01(0.00-0.08) |
| Dependence | 1 | 0.01% | 0.0122 | 0.0017 | 0.0866 | 0.01(0.00-0.09) |
| Nicotine dependence | 1 | 0.01% | 0.0128 | 0.0018 | 0.0908 | 0.01(0.00-0.09) |
| Alcoholism | 1 | 0.01% | 0.0243 | 0.0034 | 0.1726 | 0.02(0.00-0.17) |
| Hypomania | 1 | 0.01% | 0.0304 | 0.0043 | 0.2161 | 0.03(0.00-0.22) |
| Persecutory delusion | 1 | 0.01% | 0.0482 | 0.0068 | 0.3425 | 0.05(0.01-0.34) |
| Depersonalisation/derealisation disorder | 1 | 0.01% | 0.0506 | 0.0071 | 0.3592 | 0.05(0.01-0.36) |
| Impulse-control disorder | 1 | 0.01% | 0.0585 | 0.0082 | 0.4159 | 0.06(0.01-0.42) |
| Substance-induced psychotic disorder | 1 | 0.01% | 0.0644 | 0.0091 | 0.4574 | 0.06(0.01-0.46) |
| Psychiatric decompensation | 1 | 0.01% | 0.0651 | 0.0092 | 0.4627 | 0.07(0.01-0.46) |
| Schizoaffective disorder | 1 | 0.01% | 0.0684 | 0.0096 | 0.4860 | 0.07(0.01-0.49) |
| Mutism | 1 | 0.01% | 0.0791 | 0.0111 | 0.5617 | 0.08(0.01-0.56) |
| Agoraphobia | 1 | 0.01% | 0.0826 | 0.0116 | 0.5870 | 0.08(0.01-0.59) |
| Abnormal sleep-related event | 1 | 0.01% | 0.0833 | 0.0117 | 0.5916 | 0.08(0.01-0.59) |
| Morbid thoughts | 1 | 0.01% | 0.0913 | 0.0129 | 0.6489 | 0.09(0.01-0.65) |
| Sleep-related eating disorder | 1 | 0.01% | 0.1197 | 0.0168 | 0.8510 | 0.12(0.02-0.85) |
| Adjustment disorder with depressed mood | 1 | 0.01% | 0.1208 | 0.0170 | 0.8586 | 0.12(0.02-0.86) |
| Hypervigilance | 1 | 0.01% | 0.1216 | 0.0171 | 0.8641 | 0.12(0.02-0.86) |
| Disturbance in sexual arousal | 1 | 0.01% | 0.1262 | 0.0178 | 0.8973 | 0.13(0.02-0.90) |
| Negativism | 1 | 0.01% | 0.1363 | 0.0192 | 0.9691 | 0.14(0.02-0.97) |
| Soliloquy | 1 | 0.01% | 0.1420 | 0.0200 | 1.0096 | 0.14(0.02-1.01) |
| Delusional disorder, unspecified type | 1 | 0.01% | 0.1459 | 0.0205 | 1.0374 | 0.15(0.02-1.04) |
| Persistent depressive disorder | 1 | 0.01% | 0.1554 | 0.0219 | 1.1051 | 0.16(0.02-1.11) |
| Indifference | 1 | 0.01% | 0.1588 | 0.0223 | 1.1290 | 0.16(0.02-1.13) |
| Intrusive thoughts | 1 | 0.01% | 0.1657 | 0.0233 | 1.1781 | 0.17(0.02-1.18) |
| Suspiciousness | 1 | 0.01% | 0.1683 | 0.0237 | 1.1969 | 0.17(0.02-1.20) |
| Premature ejaculation | 1 | 0.01% | 0.1701 | 0.0239 | 1.2097 | 0.17(0.02-1.21) |
| Parasomnia | 1 | 0.01% | 0.1741 | 0.0245 | 1.2384 | 0.17(0.02-1.24) |
| Perinatal depression | 1 | 0.01% | 0.1754 | 0.0247 | 1.2476 | 0.18(0.02-1.25) |
| Compulsions | 1 | 0.01% | 0.1916 | 0.0269 | 1.3630 | 0.19(0.03-1.36) |
| Hallucination, tactile | 1 | 0.01% | 0.1932 | 0.0272 | 1.3741 | 0.19(0.03-1.37) |
| Grief reaction | 1 | 0.01% | 0.1968 | 0.0277 | 1.3996 | 0.20(0.03-1.40) |
| Posturing | 1 | 0.01% | 0.2039 | 0.0287 | 1.4506 | 0.20(0.03-1.45) |
| Belligerence | 1 | 0.01% | 0.2380 | 0.0334 | 1.6935 | 0.24(0.03-1.69) |
| Lack of spontaneous speech | 1 | 0.01% | 0.2518 | 0.0354 | 1.7921 | 0.25(0.04-1.79) |
| Sexually inappropriate behaviour | 1 | 0.01% | 0.2558 | 0.0359 | 1.8210 | 0.26(0.04-1.82) |
| Delusion of grandeur | 1 | 0.01% | 0.2572 | 0.0361 | 1.8309 | 0.26(0.04-1.83) |
| Delirium tremens | 1 | 0.01% | 0.2600 | 0.0365 | 1.8509 | 0.26(0.04-1.85) |
| Rapid eye movements sleep abnormal | 1 | 0.01% | 0.2666 | 0.0374 | 1.8975 | 0.27(0.04-1.90) |
| Grandiosity | 1 | 0.01% | 0.2936 | 0.0412 | 2.0908 | 0.29(0.04-2.09) |
| Trance | 1 | 0.01% | 0.2991 | 0.0420 | 2.1302 | 0.30(0.04-2.13) |
| Impaired reasoning | 1 | 0.01% | 0.3049 | 0.0428 | 2.1712 | 0.30(0.04-2.17) |
| Boredom | 1 | 0.01% | 0.3291 | 0.0462 | 2.3440 | 0.33(0.05-2.34) |
| Mental disorder due to a general medical condition | 1 | 0.01% | 0.3457 | 0.0485 | 2.4633 | 0.35(0.05-2.46) |
| Acute stress disorder | 1 | 0.01% | 0.3495 | 0.0491 | 2.4904 | 0.35(0.05-2.49) |
| Echolalia | 1 | 0.01% | 0.4113 | 0.0577 | 2.9324 | 0.41(0.06-2.93) |
| Alcohol problem | 1 | 0.01% | 0.4438 | 0.0622 | 3.1653 | 0.44(0.06-3.17) |
| Autophobia | 1 | 0.01% | 0.4970 | 0.0697 | 3.5465 | 0.50(0.07-3.55) |
| Encopresis | 1 | 0.01% | 0.5331 | 0.0747 | 3.8054 | 0.53(0.07-3.81) |
| Dissociative amnesia | 1 | 0.01% | 0.5646 | 0.0791 | 4.0319 | 0.56(0.08-4.03) |
| Excessive masturbation | 1 | 0.01% | 0.5749 | 0.0805 | 4.1052 | 0.57(0.08-4.11) |
| Illogical thinking | 1 | 0.01% | 0.5854 | 0.0820 | 4.1812 | 0.59(0.08-4.18) |
| Breath holding | 1 | 0.01% | 0.6040 | 0.0845 | 4.3143 | 0.60(0.08-4.31) |
| Malignant catatonia | 1 | 0.01% | 0.6448 | 0.0902 | 4.6078 | 0.64(0.09-4.61) |
| Histrionic personality disorder | 1 | 0.01% | 0.7284 | 0.1018 | 5.2102 | 0.73(0.10-5.21) |
| Paranoid personality disorder | 1 | 0.01% | 0.7634 | 0.1067 | 5.4622 | 0.76(0.11-5.46) |
| Fear-related avoidance of activities | 1 | 0.01% | 0.7886 | 0.1102 | 5.6443 | 0.79(0.11-5.64) |
| Personality change due to a general medical condition | 1 | 0.01% | 0.8755 | 0.1222 | 6.2712 | 0.88(0.12-6.27) |
| Alcohol use disorder | 1 | 0.01% | 1.1227 | 0.1563 | 8.0623 | 1.12(0.16-8.06) |
| Loose associations | 1 | 0.01% | 1.5904 | 0.2204 | 11.4762 | 1.59(0.22-11.48) |
| Post-traumatic amnestic disorder | 1 | 0.01% | 1.7350 | 0.2401 | 12.5379 | 1.74(0.24-12.54) |
| Hallucination, synaesthetic | 1 | 0.01% | 2.2192 | 0.3056 | 16.1160 | 2.22(0.31-16.12) |
| Alcoholic hangover | 1 | 0.01% | 2.2720 | 0.3127 | 16.5085 | 2.27(0.31-16.51) |
| Derailment | 1 | 0.01% | 2.5112 | 0.3448 | 18.2906 | 2.51(0.34-18.29) |
| Catastrophic reaction | 1 | 0.01% | 2.6507 | 0.3634 | 19.3340 | 2.65(0.36-19.33) |
| Psychogenic erectile dysfunction | 1 | 0.01% | 5.6133 | 0.7470 | 42.1806 | 5.61(0.75-42.18) |
| Vomiting psychogenic | 1 | 0.01% | 5.9641 | 0.7909 | 44.9740 | 5.96(0.79-44.97) |
| Fear of open spaces | 1 | 0.01% | 6.3617 | 0.8403 | 48.1625 | 6.36(0.84-48.16) |
| Somniphobia | 1 | 0.01% | 13.6323 | 1.6772 | 110.8047 | 13.63(1.68-110.80) |
| Emetophobia | 1 | 0.01% | 19.0852 | 2.2296 | 163.3648 | 19.09(2.23-163.36) |
